# Supplementary material for: A Critical View of the Application of the APEX Software (Aqueous Photochemistry of Environmentally-Occurring Xenobiotics) to Predict Photoreaction Kinetics in Surface Freshwaters
Source: Molecules. 2019 Dec 18;25(1):9. doi: 10.3390/molecules25010009 (PMC7017383; doi:10.3390/molecules25010009)
Supplement: Supplementary file 1 [file molecules-25-00009-s001.zip › 3.2.4_gcc-4.4.0.zip!/3.2.4_gcc-4.4.0/3.2.4_gcc-4.4.0/doc/octave/html/interpreter/Comments-and-the-Help-System.html]

Comments and the Help System - Untitled


Previous: Block Comments,
Up: Comments


---

#### 2.7.3 Comments and the Help System

The `help` command (see Getting Help) is able to find the first
block of comments in a function and return those as a documentation
string. This means that the same commands used to get help
on built-in functions are available for properly formatted user-defined
functions. For example, after defining the function `f` below,

```
     function xdot = f (x, t)
     
     # usage: f (x, t)
     #
     # This function defines the right-hand
     # side functions for a set of nonlinear
     # differential equations.
     
       r = 0.25;
       ...
     endfunction
```

the command `help f` produces the output

```
      usage: f (x, t)
     
      This function defines the right-hand
      side functions for a set of nonlinear
      differential equations.
```

Although it is possible to put comment lines into keyboard-composed,
throw-away Octave programs, it usually isn't very useful because the
purpose of a comment is to help you or another person understand the
program at a later time.

The `help` parser currently only recognizes single line comments
(see Single Line Comments) and not block comments for the initial
help text.
